# Multiple Linear Regression-Structural Equation Modeling Based Development of the Integrated Model of Perceived Neighborhood Environment and Quality of Life of Community-Dwelling Older Adults: A Cross-Sectional Study in Nanjing, China

**DOI:** 10.3390/ijerph16244933

**Published:** 2019-12-05

**Authors:** Fan Zhang, Dezhi Li

**Affiliations:** 1 Department of Construction and Real Estate, School of Civil Engineering, Southeast University, Nanjing 211189, Jiangsu, China; 2 Engineering Research Center of Building Equipment, Energy and Environment, Southeast University, Nanjing 211189, Jiangsu, China

**Keywords:** perceived neighborhood environment, quality of life, community-dwelling older adults, multiple linear regression, structural equation modeling

## Abstract

Due to the poor functioning in daily living activities, community-dwelling older adults spend more time in their neighborhood environment. The perceived neighborhood environment is crucial to their quality of life (QoL). To explore the complex influences of perceived neighborhood environment on QoL, a questionnaire was designed to measure their perception of each factor of neighborhood environment and each domain of QoL. Based on collected data, the reliability test was applied to revise the questionnaire. Multiple linear regression (MLR) and structural equation modeling (SEM) were adopted to hypothesize and test the integrated model for community-dwelling older adults. The results show that community-dwelling older adults’ perceptions of neighbor support, facilities related to physical exercise and recreation, and accessibility to facilities impact their overall QoL with diverse coefficients of 0.437, 0.312, and 0.295, respectively; neighbor support (0.207) on physical health; sidewalk condition (0.134), natural environment (0.260), and facilities related to daily life (0.165) on psychological health; and neighbor support (0.298), facilities related to daily life (0.206), and design-related safety (0.225) on social relationship. This revealed that perceptions of neighborhood environment have diverse impacts on their QoL. This study can provide targeted retrofit strategies for communities to enhance QoL of community-dwelling older adults efficiently.

## 1. Background

In recent years, the severity of the problem of society aging has been increasing worldwide. Globally, adults over 60 are expected to grow to more than 2 billion by 2050 [1]. Furthermore, the Chinese aging issue is severe. Chinese older adults will reach 0.5 billion by 2050, which will account for 36.5% of the population of China. The government must cope with this severe social issue, i.e., how to support these older adults successfully.

Moving to professional care facilities, such as nursing homes, or aging in their own communities by receiving help from family members are traditional and common situations for older adults [2]. However, their shortcomings are obvious. The expense of professional care facilities is unaffordable for many older adults, and the majority of older adults would prefer not to move to an unfamiliar environment [3]. On the other hand, if older adults choose to age in their own communities, they can continue to live in their familiar living environment, and need not pay for professional services. Instead of professional services, these community-dwelling older adults need additional supports from their family members and neighborhood. However, with the rapid development of countries, more family members spend more time at work, thus more and more older adults cannot get enough supports from family in recent years [4]. To age successfully, these community-dwelling older adults have high requirements on their living environment, i.e., communities.

People can be regarded as living and mobile within different spatial levels of their surrounding environment: micro-level, meso-level, and macro-level [5,6]. The micro-level living environment is the interior environment of residential buildings, the macro-level living environment mainly refers to the environment of the whole city/region/country, and the meso-level living environment is usually the environment within the neighborhood, which can be called neighborhood environment. Because of the functional impairment and loss of abilities, mobility, and activity, the areas of older adults decrease significantly, usually not far beyond their neighborhood [7]. Compared with the younger group, older adults spend more time in their neighborhood environment for social, recreational, and task-related activities [8]. Consequently, it is essential to improve the neighborhood environment to enhance the quality of life (QoL) of community-dwelling older adults.

### 1.1. QoL Assessments

QoL is a comprehensive and multi-dimensional concept measuring the overall well-being of an individual [9]. Experts from different fields have raised several definitions of QoL. Several famous institutions have proposed their own opinions on QoL. Mercer is one of the most famous global consulting companies. Mercer publishes an annual report called *Quality of Living City Ranking*, which can help multinational corporations to make a salary standard of different cities and provide key factors of quality of living improvement for governments. The Economist Intelligence Unit (EIU) also publishes *Quality-of-Life Index Report* and makes *Global Liveability Ranking* every year, according to their life-satisfaction survey. Mercer and EIU assess the QoL of a city, according to the economic environment, medical considerations, education, public service, and many other aspects of this city.

The QoL of the individual was first proposed by researchers in the medical field. QoL refers to individuals’ physical and psychogenic health [10]. Furthermore, individuals’ perceptions of other aspects are considered as part of their QoL, such as income, well-being, social relationship, and satisfaction, since these aspects are important components of life. The most widely used definition of QoL of the individual was proposed by the World Health Organization Quality of Life (WHOQOL) Group in 1995. WHOQOL Group defined QoL as “*individuals’ perception of their position in life in the context of the culture and value system in which they live and in relation to their goals, expectation, standards and concerns”* [11]. WHOQOL Group has developed two official versions of assessments of QoL: WHOQOL-100 published in 1995 [11] and WHOQOL-BREF published in 1996 as an abbreviated version for the easy usage [12]. It assesses the QoL by four main domains: physical health, psychological health, social relationship, and living environment. Currently, QoL has developed a systematic research field.

### 1.2. Neighborhood Environment and Older Adults

The composition of the neighborhood environment is quite complex, usually consisting of the physical aspect, natural aspect, social aspect, facilities aspect, and safety aspect. Researchers consider neighborhood environment as an important environment influencing older adults’ life. Different parts of the neighborhood environment have diverse impacts on older adults’ life. Many related studies have been conducted to identify these impacts or relations.

To the physical neighborhood environment, perception of the street connectivity is associated with the wellbeing of older adults [13]. Barrier-free design [14,15], the condition of sidewalks, the placement of crosswalk [16], and the street noise level [17] all have great influences on the QoL of older adults. Regarding the social neighborhood environment, studies have pointed out that social cohesion and social support significantly influence the wellbeing [13] and QoL [18,19] of older adults. Moreover, the percent poverty, residential stability, and the concentration of older adults within the neighborhood influence self-rated health of older adults [20]. The peer support [7,21] and percent extreme poverty [22] are closely related to physical activities of older adults. Especially, depressive symptoms of older adults are affected heavily by the social support networks within the neighborhood [23].

The natural environment problem is a key factor in the life satisfaction of older adults [24]. Air pollution significantly influences the mental health of the elderly [25]. The facility with the neighborhood is also a curial aspect for older adults. Security facilities, communal facilities, medical facilities, care facilities, outdoor space, and activities centers for older adults have close associations with the wellbeing [26] and satisfaction of older adults [27]. Furthermore, service accessibility [27], environmental accessibility [28], and parks [21] are found to affect physical activities of older adults heavily. For older adults with a slower rate of cognitive decline, community resources, proximity to public transit, and public spaces are key influencing factors of their mental health [29]. For older adults with depressive symptoms, perceived accessibility of facilities also affects their depressive symptoms heavily [23]. In safety aspect of neighborhood environment, neighborhood safety affects physical activities [7] and the overall QoL [18] of older adults. Security [16] is closely associated with the QoL of older adults. Health-related QoL of older adults is heavily influenced by the safety from traffic [17] and crime [14].

However, there are also studies holding opposite opinions. For instance, the built environment within the neighborhood [30] and the accessibility to green spaces or senior centers [31] are considered to have a non-significant association with self-rated health of older adults.

According to the review, many previous studies have proposed and analyzed diverse influence relations between neighborhood environment and older adults’ QoL. Most studies have checked influence relations between each factor of the neighborhood environment and each domain of older adults independently, while few studies have integrally considered all possible influences of perceived neighborhood environment on the QoL of community-dwelling older adults, and an integrated model is lacking to reflect these complex influences.

Consequently, this study aimed to develop the integrated model for community-dwelling older adults, in order to consider all possible influences of their perceived neighborhood environment on their QoL. A questionnaire was designed based on the components of urban neighborhood environment and domains of QoL to collect perception data from community-dwelling older adults. Based on the questionnaire, two surveys were conducted. Data from the first-round survey with a smaller scale were analyzed by reliability test for revising the questionnaire and the multiple linear regression (MLR) analysis for identifying the significant influences and raising the basic hypothesis of the integrated model for community-dwelling older adults. The second-round survey with a larger scale was applied to verify the hypothesis of the integrated model through the structural equation model (SEM). This integrated model provides valuable retrofit strategies of neighborhood environment to improve QoL of community-dwelling older adults more efficiently.

## 2. Materials and Methods

### 2.1. Questionnaire Design

**Figure 1:**
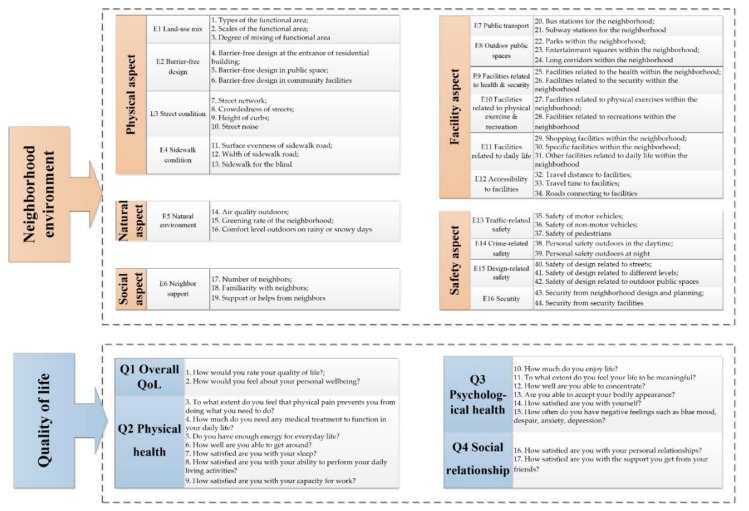
Descriptive items of perceived neighborhood environment and QoL.

Based on the literature review, the neighborhood environment where older adults live contains 16 factors significantly affecting QoL of older adults [32]. These factors belong to five aspects of the neighborhood environment. The physical aspect of neighborhood environment refers to artificial components of tangible environment, containing land-use mix (E1), barrier-free design (E2), street condition (E3), and sidewalk condition (E4). The natural aspect of neighborhood environment is the nature and ecology within the neighborhood, including only natural environment (E5). The social aspect of neighborhood environment includes social support (E6). The facility aspect of neighborhood environment means facilities providing the essential services for older adults, containing public transport (E7), outdoor public spaces (E8), facilities related to health and security (E9), facilities related to physical exercise and recreation (E10), facilities related to daily life (E11), and accessibility to facilities (E12). The safety aspect of neighborhood environment contains traffic-related safety (E13), crime-related safety (E14), design-related safety (E15), and security (E16). Since these factors are professional concepts which may be hard to understand by many older adults, their perception of factors of the neighborhood environment cannot be measured directly. Thus, several descriptive items were designed to describe the perception with neighborhood environment in detail, which have already been validated [33]. Neighborhood environmental factors and descriptive items are shown in Figure 1. For instance, the factor of “street condition” is measured by four descriptive items: street network, crowdedness of streets, height of curbs, and street noise. Overall, there are 44 descriptive items belonging to 16 neighborhood environmental factors. The five-point Likert scale was applied to quantize their satisfaction level, ranging from 1 (very unsatisfied) to 5 (very satisfied).

According to WHOQOL-BREF [12], QoL of community-dwelling older adults was assessed in four domains—(Q1) overall QoL, (Q2) physical health, (Q3) psychological health, and (Q4) social relationship—using 17 descriptive items to describe the four domains of QoL. Domains of QoL and descriptive items are shown in Figure 1. For example, the overall QoL of older adults was assessed by two descriptive items: “*How would you rate your quality of life?*” and “*How would you feel about your personal wellbeing*”. All the items were scored based on the steps of WHOQOL-BREF.

### 2.2. Data Analysis

As mentioned above, descriptive items of factors of perceived neighborhood environment have been validated by “NE-QoL” model [33], and descriptive items of domains of QoL have been validated by WHO [12]. Thus, it was not necessary to conduct a validation test, but the reliability test was still essential to check the reliability of the questionnaire. Analysis data were obtained from the first-round survey with the original questionnaire. The reliability test is usually conducted through calculating the Cronbach’s alpha value (*α-*value), which is regarded as the indicator to present the reliability of each descriptive item. The computational formula is as follows.

(1) $$\alpha =(\frac{k}{k-1})*(1-\frac{\sum S_{i}{}^{2}}{S_{T}{}^{2}})$$

where *k* is the number of descriptive items in the questionnaire, *S_i_^2^* is the variance of factors and domains, and *S_T_^2^* is the variance of descriptive items.

Generally speaking, if the *α-*value of one factor or one domain is greater than 0.6, descriptive items of this factor or domain in the questionnaire are appropriate and can be accepted; if *α-*value of one factor or one domain is greater than 0.8, descriptive items of this factor or domain in questionnaire are considered to be designed very well; and, if *α-*value of one factor or one domain is less than 0.6, it indicates descriptive items of this factor or domain need to be modified [34]. Besides, “*α-*value if deleted” is another important indicator that shows the final *α-*value if this descriptive item is deleted from the original questionnaire. If “*α-*value if deleted” is much higher than the *α-*value, it indicates this descriptive item has great negative effects on the reliability of this factor or domain, and this item should be removed from the questionnaire.

The MLR analysis is used to identify the significant linear influence relations between factors of perceived neighborhood environment and domains of the QoL of community-dwelling older adults through stepwise regression. The final value of perceived neighborhood environmental factors and domains of QoL is the average of its descriptive values. The MLR analysis was conducted using the Statistical Package of Social Science (SPSS) version 22.0. However, MLR analysis can only test direct linear relations between several factors of perceived neighborhood environment and one domain of QoL once, thus MLR analysis cannot consider all domains of QoL at the same time.

After identifying significant linear influence relations, the hypothesis of the integrated model for community-dwelling older adults, which contain all possible significant influences, can be proposed. The SEM includes a diverse set of mathematical models, computer algorithms, and statistical methods to constructs relationships between variables based on collected data. SEM was chosen to verify this hypothesis because: (1) as an extension of linear regression model, SEM allows for complex influences between perceived neighborhood environment and QoL at one time; (2) SEM allows measurement errors of variables, thus it is suitable for unobservable variables, such as factors of perceived neighborhood environment and domains of QoL that need to be assessed by several descriptive items; and (3) SEM can provide overall fit indices to verify the matching rate between data and the hypothetical model, thus it can explain the integrated model better [35,36]. The factors of perceived neighborhood environment and domains of QoL were regarded as latent variables that cannot be measured directly, while the descriptive items were regarded as observed variables. However, the requirement of the sample size of SEM was larger than the reliability test and the MLR analysis, thus the second-round survey with a larger scale was necessary to collect enough data for SEM. To improve the efficiency of the survey, the questionnaire of the second-round survey only needed to contain descriptive items of factors of perceived neighborhood environment and domains of QoL that exist in the hypothesis of the integrated model. The SEM was conducted by using the Analysis of Moment Structure (AMOS) version 21 (IBM, NY, United States).

**Figure 2:**
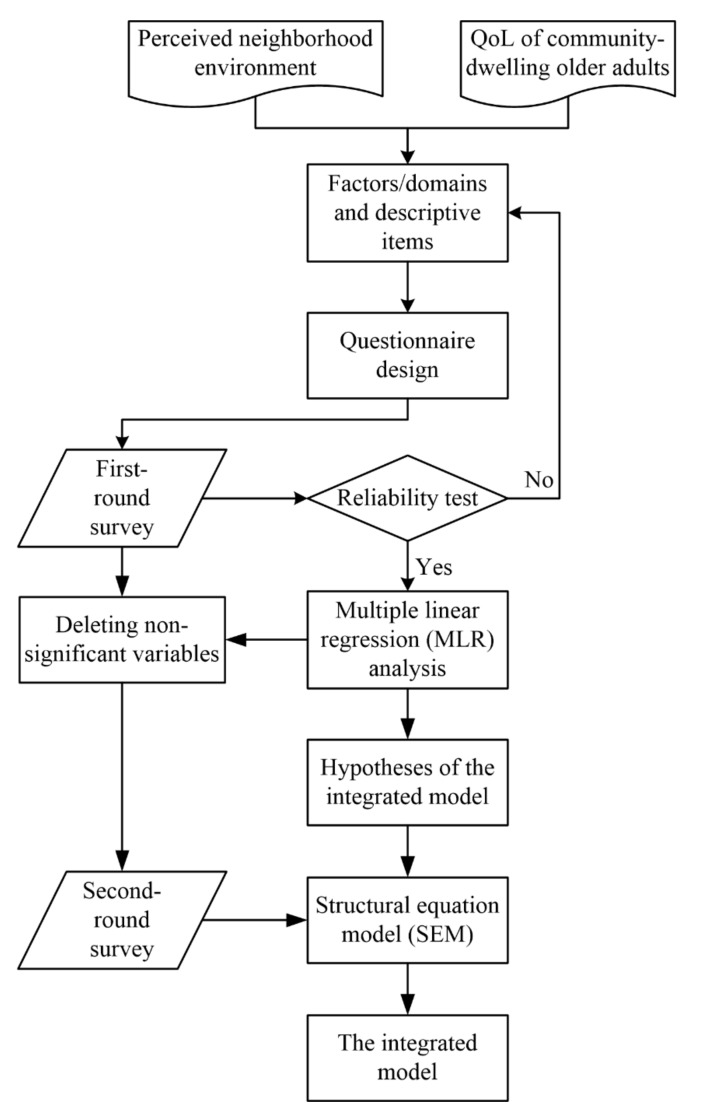
The research flow of this study.

The whole research flow is presented in Figure 2.

### 2.3. Sampling and Data Collection

First, the sampling criteria should be set to obtain appropriate research data. Since this study focused on community-dwelling older adults, only older adults who meet following inclusion criteria were sampled as eligible respondents: (1) over 60 years old; (2) live in a community; and (3) can communicate and express their perception well.

Surveys were conducted during September–October 2018 in Nanjing, and cross-section data were collected twice from the questionnaire survey based on the same sampling criteria. The survey aimed to obtain community-dwelling older adults’ perception of the neighborhood environment where they live and their QoL when they live in this neighborhood. The initial questionnaire was only designed based on descriptive items of neighborhood environment and QoL. The three sections in the initial questionnaire regarded the general information of respondents (including age, gender, type of community, who they live with, and length of residence), the perception of neighborhood environment, and the QoL assessment. The first-round survey collected data with the initial questionnaire. In total, 204 qualified community-dwelling older adults participated, and 192 valid responses were obtained with a response rate of 94.12%. All procedures performed in this study involving human participation were with ethics approval from an independent research ethics committee of Southeast University. All participants were informed about the purpose of the study and their right to refuse participation or terminate their involvement during the study and informed consent were obtained.

Then, the questionnaire was modified according to the results of the reliability test. Only the perceived neighborhood environmental factors that show significant relations with QoL of community-dwelling older adults were kept in the questionnaire, and the others were deleted from the initial questionnaire. The second-round survey with modified questionnaire was conducted on a larger scale, collecting 455 responses. Table 1 shows the general information of respondents. Overall, 49% of respondents are female and 51% are male, while 65% of older adults are 60–70, 15% are 70–80, and 20% are over 80 years old.

## 3. Results

### 3.1. Results of Reliability Test of the Questionnaire

**Figure 3:**
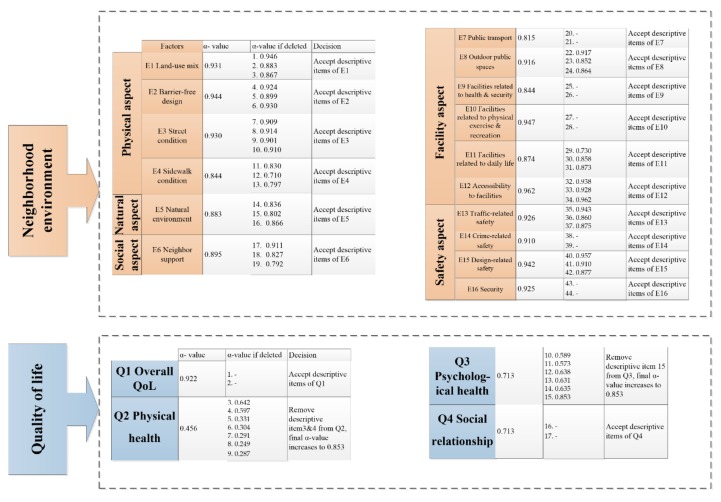
The reliability test of factors of perceived neighborhood environmental factors and domains of QoL of community-dwelling older adults.

According to data analysis, the reliability test needed to be conducted to ensure the internal consistency of descriptive items in the questionnaire. The results of the reliability test of factors of perceived neighborhood environment and domains of QoL of community-dwelling older adults are shown in Figure 3.

In the orange part of Figure 3, *α-*value and “*α-*value if deleted” are the main indicators of the reliability test of descriptive items of perceived neighborhood environment. Obviously, the *α-*values of all factors are higher than 0.8, meaning that descriptive items of all factors were designed well for this study. Furthermore, each “*α-*value if deleted” was not much higher than its respective *α-*value. This means that deleting any descriptive items from the questionnaire cannot increase the results greatly, and the detailed items of perceived neighborhood environment did not need modification.

Figure 3 shows the reliability of domains of QoL in the blue part. The internal consistency of domains of physical health and psychological health are poor. There are seven descriptive items in the domain of physical health, and the *α-*value of the domain of physical health is only 0.456, less than 0.6. Since the “*α-*values if deleted” of Items 3 and 4 are much higher than their *α-*values, deleting Items 3 and 4 from the domain of physical health would increase the *α-*value to 0.853. The *α-*value of the domain of psychological health is higher than 0.6, but deleting Item 15 can heavily improve the *α-*value to 0.853. Finally, Items 3, 4 and 15 should be removed from descriptive items of QoL of community-dwelling older adults.

### 3.2. Results of MLR Analysis

Based on data from the first survey, MLR analysis was conducted to investigate the correlation between factors of perceived neighborhood environment and domains of QoL. Table 2 shows the results of MLR analysis in detail. It is easy to find that the overall QoL of community-dwelling older adults is affected by E6, E10, and E12 significantly, and community-dwelling older adults’ perception of these perceived neighborhood environmental factors explain 46.2% of the variance of their overall QoL. The physical health of community-dwelling older adults is only meaningfully influenced by their perception of E6, and 10.5% of its variance can be explained. Community-dwelling older adults’ perception of E11, E4, and E5 impact on their psychological health significantly, explaining 26.3% of the variance. The perceptions of E6, E11, and E15 are the main factors influencing the social relationship of community-dwelling older adults, explaining 29.7% of the variance of their social relationship.

### 3.3. Hypothesis of the Integrated Model

**Figure 4:**
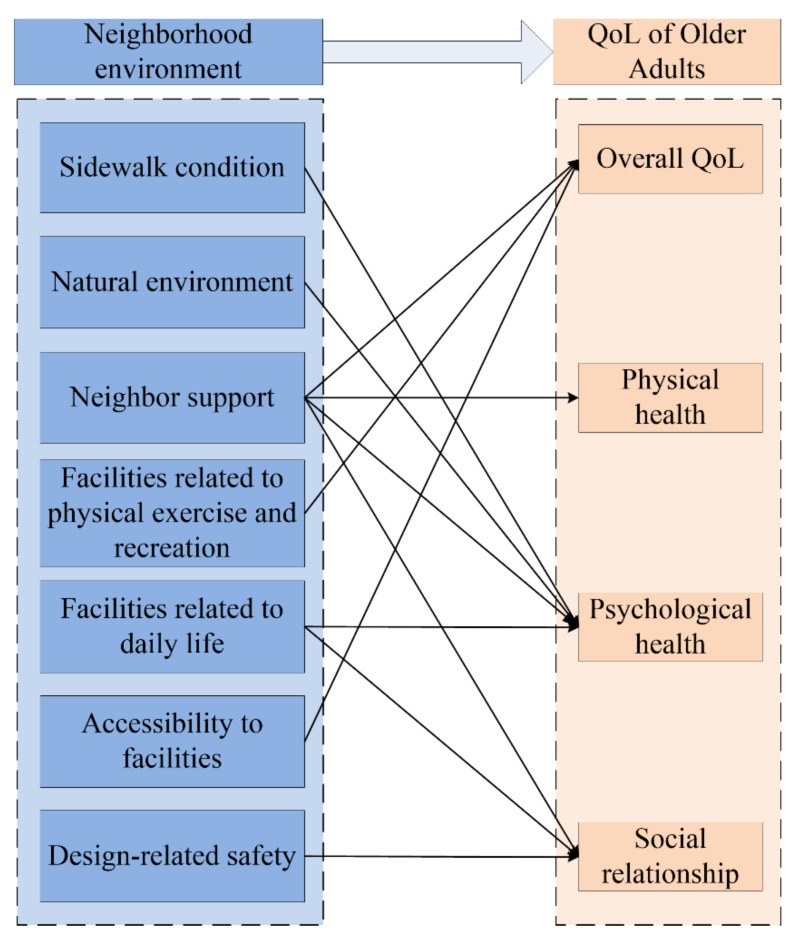
The hypothesis of the structure of the integrated model.

The results of MLR analysis present the significant influences of particular factors of perceived neighborhood environment on each domain of QoL of community-dwelling older adults. Based on these results, factors of perceived neighborhood environment may have diverse impacts on different domains of QoL of community-dwelling older adults, and it is reasonable to propose the hypothesis of the integrated model for community-dwelling older adults. All significant influences in results of MLR analysis can be assumed in the integrated model, and the detailed structure of the hypothesis of this integrated model is shown in Figure 4.

### 3.4. Results of SEM of the Integrated Model

**Figure 5:**
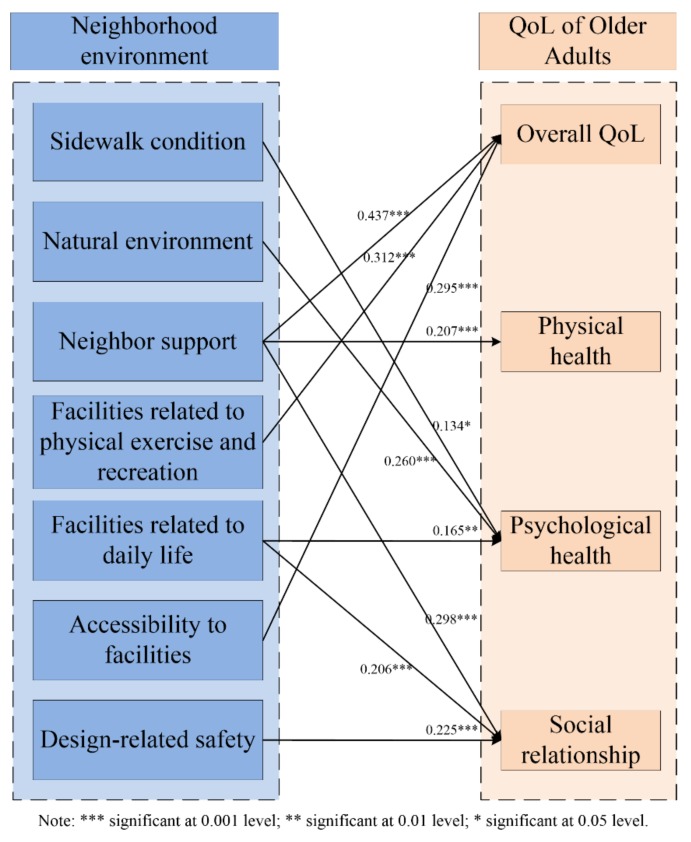
The integrated model for community-dwelling older adults.

The integrated model for community-dwelling older adults was verified by SEM analysis, which was conducted with data collected from the second-round survey. Table 3 shows fit indices of the integrated model for community-dwelling older adults and the acceptable range of these fit indices [36,37]. All fit indices are in the acceptable range, indicating the integrated model is an acceptable model fit of the data. Table 4 presents path parameters of the integrated model. The final integrated model for community-dwelling older adults is established in Figure 5.

## 4. Discussion

According to the results of MLR and SEM analyses, several factors of perceived neighborhood environment influencing the QoL of community-dwelling older adults significantly are identified. However, the impacts of perceived neighborhood environment vary with detailed factors. For more clearly, factors of perceived neighborhood environment were divided into five aspects: physical aspects, natural aspects, social aspect, facilities aspect, and safety aspect. The main findings explored from the results are as follows.

### 4.1. Physical Aspect

The physical aspect of the perceived neighborhood environment is quite important to the whole neighborhood environment, which refers to artificial components of the tangible environment. E1–E4 are four key factors belonging to this physical aspect of perceived neighborhood environment. Nevertheless, only sidewalk condition impacts on the psychological health of community-dwelling older adults significantly. It is an interesting finding. The sidewalk is an important transportation infrastructure where older adults can walk [38]. Compared with other walkable transportation infrastructures, such as roads or trails, community-dwelling older adults rely more on the sidewalk. The main reason may be that the “pedestrian-and-vehicle dividing system” is still not widely applied in a certain proportion of communities in China. Many community-dwelling older adults need to walk outdoors every day, and, without the “pedestrian-and-vehicle dividing system”, they have to walk along the sidewalk [39]. A better sidewalk condition with an even surface, enough width, and sidewalk for the blind has positive impacts on their sense of control [16], makes them feel more relax, and benefits their psychological health. Thus, the sidewalk condition should be attended to if older adults are to be more active physically [5].

### 4.2. Natural Aspect

The natural aspect of perceived neighborhood environment refers to the natural environment (E5) within the neighborhood, including air quality outdoors, community afforestation, and environment on rainy and snowy days. According to the integrated model for community-dwelling older adults (Figure 5), the natural aspect of perceived neighborhood environment has impacts only on their psychological health distinctly [25]. Generally, natural neighborhood environment is considered to affect the physical health of older adults, since community-dwelling older adults may feel uncomfortable under a poor natural neighborhood environment. Supplementary in-depth interviews with community-dwelling older adults were conducted. Many community-dwelling older adults living in urban communities prefer staying at home and reducing going out when natural neighborhood environment is bad, for example, unhealthy air quality, cold and snowy weather, etc. Therefore, a worse natural environment would not influence physical health of community-dwelling older adults heavily, but can reduce their outdoor activity opportunities and prevent them from enjoying life, and thus lower their psychological health.

### 4.3. Social Aspect

In this study, the social aspect of perceived neighborhood environment mainly refers to neighbor support (E6), which is the support that community-dwelling older adults obtain from their neighbors. As shown in Figure 5, neighbor support is a crucial influence factor of the neighborhood environment, since neighbor support has a significant influence on the overall QoL, the physical health, and the social relationship of community-dwelling older adults.

Neighbor support generally includes two types: formal neighbor support and informal neighbor support [7]. Formal neighbor support means organized help or communication within neighborhood, for instance the team of running or dancing and the team of peer supports for older adults, and these teams would organize formal activities regularly. Informal neighbor support refers to help or communication within the neighborhood, which usually happens casually, such as meeting or talking with neighbors by chance, and helping neighbors carry heavy goods. The integrated model for community-dwelling older adults reveals that neighbor support affects the physical health, the social relationship, and the overall QoL of community-dwelling older adults. It is easy to understand that neighbors are an important part of the social relationship of older adults, so neighbor support is crucial to the social relationship of community-dwelling older adults. Both formal and informal support can provide opportunities to become familiar with neighbors and obtain support. More neighbor support would prevent older adults from physical injuries or sudden diseases. Community-dwelling older adults express that neighbors usually help them when they have difficulty walking, when they fall, or when they are in an emergency such as a sudden heart attack. Finally, neighbor support makes community-dwelling older adults feel their daily life interesting and convenient, thus neighbor support also has a positive impact on their perception of overall QoL.

### 4.4. Facility Aspect

The facility aspect of the perceived neighborhood environment contains the facilities located within the neighborhood that provide the essential services for community-dwelling older adults, including E7–E12. Among factors of the facility aspect, only facilities related to physical exercise and recreation, facilities related to daily life, and accessibility to facilities affect the QoL of community-dwelling older adults significantly.

Facilities related to physical exercise and recreation are places where community-dwelling older adults can do physical exercises or recreation. Many community-dwelling older adults consider that their physical health is mainly determined by illnesses and injuries, thus facilities related to physical exercises cannot improve their physical health directly and significantly. However, more exercise can improve their individual perceptions of their overall QoL [40]. Facilities related to recreation, such as community libraries and senior centers, provide places where community-dwelling older adults can have fun and enjoy their lives, increasing their personal wellbeing.

The facilities related to daily life are crucial to community-dwelling older adults, since they provide essential services supporting their daily lives [41]. Facilities related to daily life include those where older adults can shop, e.g., shopping malls, CVS, supermarkets, etc.; those that provide specific services for older adults, e.g., bookstores, laundries, banks, etc.; and those providing other essential services, e.g., delivery lockers, parking lots, etc. The allocation of facilities related to daily life within the neighborhood can improve the psychological health of community-dwelling older adults by relieving their stress of necessities purchase and services obtainment every day. Besides, the social relationship of community-dwelling older adults is also enhanced. Many community-dwelling older adults explained that, if facilities related daily life with a good enough quality were located within the neighborhood, most of the residents would like to use services of these facilities, and then they can often meet many neighbors at facilities, increasing opportunities to get familiar with neighbors.

Another factor of the facility aspect is the accessibility to facilities. The accessibility to facilities shows how difficult it is to arrive and obtain services in facilities for community-dwelling older adults, and there is not yet a unified definition for assessment [42]. For instance, relative accessibility and integrated accessibility is assessed based on space separation factors such as distances and time [43], and the two-step floating catchment area method [44] calculates accessibility based on the cumulative-opportunities. In the integrated model, accessibility to facilities impacts on the perception of the overall QoL heavily. Older adults with higher accessibility to facilities can obtain necessities and services more easily, and they have fewer difficulties in maintaining their lives. Therefore, accessibility to facilities can improve the living satisfaction and overall QoL of community-dwelling older adults [24].

### 4.5. Safety Aspect

E13–E16 are four factors belonging to safety aspect of the perceived neighborhood environment. Among the four factors of the safety aspect, only design-related safety has a significant influence on the social relationship of community-dwelling older adults. Design-related safety means residents’ sense of feeling physically safe because of the neighborhood design [7]. Based on the in-depth interviews, if the neighborhood design is quite safe for older adults, they would feel free to move within the neighborhood environment. For instance, community parks are designed safely with signage, handrails, soft ground, appropriate lighting, etc. Since the design guarantees safety, community-dwelling older adults can perceive better wellbeing and overall QoL.

## 5. Conclusions and Implications

This study explored how the perceptive neighborhood environment affects the QoL of community-dwelling older adults, pointed out significant influences between perceived neighborhood environment factors and domains of QoL of community-dwelling older adults, and then developed the integrated model for community-dwelling older adults. The integrated model reveals that at least one factor belonging to each aspect of urban neighborhood environment— containing physical aspect, natural aspect, social aspect, facility aspect and safety aspect—significantly influences the QoL of community-dwelling older adults, but influence relations vary with the aspects of perceived neighborhood environment and domains of QoL.

Consequently, the practical implication of this study is to provide valuable retrofit strategies for neighborhood environment to enhance the QoL of community-dwelling older adults. When government and community managers consider retrofitting communities, they can conduct a sample survey about QoL of older adults. With the feedback from older adults from each community, government and community managers can propose targeted retrofit strategies of neighborhood environment for different communities according to the integrated model. For instance, if the feedback from older adults living in the community presents lower psychological health status, the government and community managers should pay more attention to sidewalk condition and the natural environment and facilities related to daily life during retrofitting the community. However, since respondents of this study are from different regions of China, regional differentiation of the neighborhood environment may influence the final integrated model. The following study will explore the inter-regional differences of influence relations between the neighborhood environment and the QoL of community-dwelling older adults.

## Figures and Tables

**Table 1:** General information of respondents.

General Information of Respondents	Options	Frequency	Percentage
Age	60–69	296	65.05%
	70–79	68	14.95%
	≥80	91	20.00%
Gender	Female	223	49.01%
	Male	232	50.99%
Type of community	Commercial housing	245	53.85%
	Affordable housing	125	27.47%
	Self-built housing	69	15.16%
	Others	16	3.52%
Who they live with	Live alone	38	8.35%
	Live with partner	284	62.42%
	Live with children	117	25.71%
	Others	16	3.52%
Length of residence	≤5 years	112	24.62%
	5–10 years	109	23.96%
	≥10 years	234	51.42%

**Table 2:** Results of MLR analysis.

Regression Equation		B	S.E.	T	Sig.T	R	R^2^	F	Sig.
Q1 Overall QoL	Constant	1.032	0.208	4.970	0.000	0.679	0.462	53.743	0.000
	E6	0.384	0.073	5.244	0.000				
	E12	0.226	0.072	3.125	0.002				
	E10	0.123	0.060	2.085	0.038				
Q2 Physical health	Constant	2.454	0.232	10.581	0.000	0.324	0.105	22.538	0.000
	E6	0.301	0.064	4.728	0.000				
Q3 Psychological health	Constant	0.394	0.473	0.833	0.406	0.512	0.263	16.640	0.000
	E11	0.324	0.065	4.959	0.000				
	E6	0.151	0.075	2.009	0.046				
	E4	0.254	0.075	3.410	0.001				
	E5	0.187	0.078	2.386	0.018				
Q4 Social relationship	Constant	0.444	0.531	0.836	0.404	0.545	0.297	26.450	0.000
	E6	0.466	0.075	6.183	0.000				
	E11	0.268	0.075	3.577	0.000				
	E15	0.205	0.087	2.359	0.019				

Notes: B, unstandardized coefficients; S.E., standard error; Sig., significance.

**Table 3:** Fit indices of the integrated model for community-dwelling older adults.

Fit Indices	Acceptable Range	Measured Value
df	−	496
x^2^	−	1253.404
x^2^/df	<3	2.527
GFI	>0.8	0.846
AGFI	>0.8	0.836
CFI	>0.9	0.921
RMSEA	<0.08	0.058
NNFI	>0.9	0.910
IFI	>0.9	0.921

**Table 4:** Path parameter estimates of the integrated model for community-dwelling older adults.

Path	Beta	B	S.E.	T	Sig.
Psychological health ← Sidewalk condition	0.134	0.097	0.041	2.353	*
Psychological health ← Natural environment	0.260	0.159	0.036	4.369	***
Overall QoL ← Neighbor support	0.437	0.471	0.061	7.771	***
Social relationship ← Neighbor support	0.298	0.327	0.075	4.356	***
Overall QoL ← Facilities related to physical exercise and recreation	0.312	0.325	0.053	6.079	***
Psychological health ← Facilities related to daily life	0.165	0.105	0.038	2.793	**
Physical health ← Neighbor support	0.207	0.185	0.047	3.970	***
Overall QoL ← Accessibility to facilities	0.295	0.311	0.055	5.649	***
Social relationship ← Facilities related to daily life	0.206	0.192	0.054	3.588	***
Social relationship ← Design-related safety	0.225	0.209	0.060	3.485	***

Notes: Beta, standardized coefficients; B, unstandardized coefficients; S.E., standard error; Sig., significance; *** significant at 0.001 level; ** significant at 0.01 level; * significant at 0.05 level.
